# Trauma symptoms and their correlates among Zimbabwean adolescents: Baseline results from a cluster randomized controlled trial

**DOI:** 10.1371/journal.pmen.0000719

**Published:** 2026-09-09

**Authors:** Colleen M. Fisher, Roswitta Gatsi, Natasha Venables, Regina Banda, Sarah Moore, Anastasia Hartzes

**Affiliations:** 1 Present affiliation: Graduate School of Social Work, University of Denver, Denver, Colorado, United States of America; 2 Affiliation where work was carried out: Department of Social Work, University of Alabama at Birmingham, Birmingham, Alabama, United States of America; 3 Department of Early Childhood and Junior Education, Faculty of Education, University of Zimbabwe, Harare, Zimbabwe; 4 Community Development Consultant, Harare, Zimbabwe; 5 Faculty of Social and Behavioral Sciences, University of Zimbabwe, Harare, Zimbabwe; 6 Department of Social Work, University of North Texas, Denton, Texas, United States of America; 7 Department of Biostatistics, University of Alabama at Birmingham, Birmingham, Alabama, United States of America; PLOS: Public Library of Science, UNITED KINGDOM OF GREAT BRITAIN AND NORTHERN IRELAND

## Abstract

Adolescent mental health is an urgent global priority, yet services remain severely inaccessible throughout Southern Africa. Zimbabwean youth are particularly vulnerable to mental health challenges from adverse childhood experiences including food insecurity, sexual and emotional violence, climate change-induced disasters, and national economic difficulties but research is extremely limited. To address this gap, this study examined trauma symptoms and resilience among a large school-based sample of urban and rural Zimbabwean adolescents. A cluster randomized controlled trial was conducted to evaluate the impact of *Singing to the Lions*, a biopsychosocial workshop for trauma-affected youth in Zimbabwe. Adolescents were randomized by school (n = 13) to an immediate intervention arm or waitlist control arm. Baseline data, including adolescents’ demographics, trauma symptoms, and resilience characteristics were analyzed to investigate the prevalence of trauma symptoms and that association with resilience characteristics. The baseline sample (n = 1,342) ranged in age from 12-18 years $(\overset{―}{x}$=14.8); the majority of children were girls (54.6%) and rural (52.4%). Of 18 possible trauma symptoms, youth endorsed an average of 7.6 trauma symptoms (*SD* = 4.5) with significantly higher rates among girls (*t*[1325]=3.39, *p* = .0007). Trauma symptoms were significantly inversely correlated wi*t*h resilience characteristics including self-esteem (*r* = -.36, *p* < .001), hope (*r* = -.07, *p* = .01), self-calming (*r* = -.30, *p* < .001), and social support (*r* = -.23, *p* < .001); however, there was no association between symptoms and self-efficacy. No statistically significant differences in trauma symptom prevalence were found based on children’s age, grade, or community (rural versus urban). The high prevalence of trauma symptoms and expressions of trauma found in this study are consistent with the limited previous adolescent mental health research from Sub-Saharan Africa. Results contribute to the sparse knowledge base on trauma-affected youth and adolescent mental health in Zimbabwe more broadly and highlight the need for evidence-based practices that are culturally grounded and contextually specific for the region.

## Introduction

The mental health of children and adolescents is an urgent global concern with mental disorders affecting one in seven youth [1,2]. Exposure to adverse childhood experiences (ACEs) such as poverty, abuse, and violence significantly increase vulnerability to poor mental health outcomes including the impairment of emotion regulation and resilience as well as post-traumatic stress disorder, depression, and substance use disorders [3–6]. Children and adolescents in low- and middle-income countries (LMIC) face increased vulnerability to mental health challenges due to heightened exposure to adverse childhood experiences [7–9]. Yet, despite this heightened risk, mental health services remain underfunded and difficult to access. Government spending on mental health in this region remains below 1% of national health budgets, and the region faces a severe shortage of mental health professionals, with only 0.05 to 1.52 psychiatrists per 100,000 people, compared to 9.9 per 100,000 in Europe [10].

### Causes of trauma among Zimbabwean youth

Trauma is defined as the lasting adverse effects resulting from events or circumstances experienced as physically or emotionally harmful or life-threatening [11]. In this study, experiences such as abuse, violence, neglect, household dysfunction and exposure to adversity are conceptualized as potentially traumatic experiences that may contribute to the development of trauma symptoms and related mental health difficulties. This conceptualization is consistent with research demonstrating that adverse childhood experiences, including abuse, neglect, and household dysfunction, are associated with increased risk of poor mental and physical health outcomes [12,13].

Trauma is highly prevalent in Zimbabwe, particularly among women, girls, and young children. Zimbabwe’s Violence Against Children Surveys (VACS), conducted in 2010 and 2017, revealed a high prevalence of physical, sexual, and emotional violence (e.g., being humiliated in front of others or being threatened with abandonment) [14]. Moreover, 64.1% of children experienced violent discipline such as slapping, beating, hitting with an object, or threatening a child with a weapon by a parent, caregiver, or other adult [15–17].

Emotional violence, often a precursor to physical abuse, is pervasive in family settings [18]. Additionally, historical and socio-economic factors—including the HIV/AIDS epidemic, Zimbabwe’s prolonged economic crisis (2001–2009), food insecurity, and climate change-induced disasters—contribute to widespread post-traumatic stress [19]. A recent study of Zimbabwean adolescents found high rates of mental distress among youth who had been exposed to sexual violence, interpersonal violence, community violence, physical violence, and/or orphanhood [20]. Over one-fourth of the sample reported mental distress that was moderate or severe, with psychological distress increasing significantly among youth with exposure to multiple ACEs [20]. Gender may also play an important role in understanding the causes of trauma for Zimbabwean youth, although research is extremely limited. A study of adolescents in Malawi, however, found that boys were more likely to experience physical and emotional abuse, while girls faced higher risks of sexual violence [21].

While high ACE exposure weakens one’s coping capacity and increases the risk of long-term psychopathology, psychological resilience can buffer trauma’s effects and determine how youth adapt to and recover from adverse childhood experiences [22]. Enhancing resilience, or the mental process through which a young person negotiates, manages, and adapts to trauma or other significant stressors [23], thus offers a potential strategy for strengthening the mental health of youth in high-ACE contexts. However., the specific role of resilience remains underexplored [24],and research on resilience among youth in sub-Saharan Africa is extremely limited [25,26].

### Trauma symptoms among adolescents

Traumatic experiences in childhood and adolescence often are followed by a manifestation of similar symptoms that have been observed across geographic borders as well as types of trauma [27–31]. Post Traumatic Stress Disorder (PTSD) often develops after a person experiences, witnesses, or learns about a traumatic event involving actual or threatened death, serious injury, or sexual violence. PTSD is a clinically assessed combination, pattern and timing of symptoms following a trauma that can include recurrent and distressing memories, repetitive dreams, intense physiological reactions when reminded of the trauma, and persistent avoidance of thinking, talking or going to places that remind one of the trauma [32]. Survivors diagnosed with PTSD may have feelings of worthlessness, feelings of culpability, flashbacks, an exaggerated startle response, and difficulty focusing or sleeping. The fear of recurrence and trauma-related guilt are two key symptoms in adolescents. Adolescent trauma survivors may also be particularly prone to challenges in daily functioning, including peer, school, and family interactions [30].

Young people in low-resource contexts whose communities do not routinely screen for PTSD may go undiagnosed while continuing to experience trauma-related symptoms. Further, there is emerging research suggesting that the symptoms of PTSD may manifest differently in Southern African contexts compared to Western countries from which the vast majority of research on PTSD is derived. A study of South African and Kenyan youth utilizing a Child PTSD Checklist found that the most frequently reported symptoms were avoidance of activities, places or people associated with the trauma; avoidance of thoughts, feelings or conversations associated with the trauma; irritability or outbursts of anger; and intense psychological distress at exposure to traumatic reminders [33]. Using the same instrument, researchers reported that South African youth who experienced multiple traumatic events had more symptoms of PTSD and depression than those who were exposed to a single traumatic event [34]. Another study assessed the impact of trauma on mental distress among Zimbabwean youth using the Kessler-6 (K-6), a nonspecific psychological scale assessing whether youth had felt nervous, hopeless, restless, so sad that nothing could cheer them up, that everything was effortless, or feel worthless about everything [20]. The study found higher rates of mental distress for girls compared to boys, and for older adolescents compared to their younger counterparts.

Noting the potential disconnect between Western assessment tools frequently used in trauma research and African contexts, Murray and colleagues [35] explored the applicability of the UCLA Child Post-traumatic Stress Disorder—Reaction Index with Zambian youth [30]. Researchers adapted the PTSD-RI by incorporating culture-specific information, resulting in a “locally validated” 18-item instrument with locally relevant terminology describing youths’ trauma symptoms (e.g., “thinking too much” and “having an unsettled mind”) that could be used alone or combined with the original PTSD-RI instrument for a 38-item assessment. Results indicated that the trauma symptom scale using local language to describe symptoms was effective for identifying Zambian youth who had experienced trauma and was recommended for early identification of mental health problems in youth. Using this adapted instrument, Familiar and colleagues found high rates of trauma symptoms with an average of 21.6 symptoms (out of 38) endorsed by both males and females [36]. Although these limited studies cannot be generalized, they do highlight the need for more research into the ways in which the trauma experiences of youth in low resource contexts may be shaped by cultural and contextual factors that must be understood in order to develop effective and culturally relevant mental health interventions.

Collectively, these studies suggest substantial unmet mental health needs in the Southern Africa region, comparatively little research exists on trauma and other aspects of mental health among youth in Zimbabwe. Highlighting this knowledge gap, the United Nations Children’s Fund (UNICEF) reported that the mental health of Zimbabwean children and adolescents is “underreported, suffers from lack of reliable data, remains stigmatized, and is rarely acknowledged” [37]. Recently published studies have contributed substantially to understanding ACEs among Zimbabwean adolescents and associated risks including suicide, violence, and sexually transmitted infections and lay a strong foundation for understanding mental health by using a nationally representative sample [20,38,39]. However, because the studies all used data from the 2017 Violence Against Children and Youth Survey, they offer limited insights into the current mental health experiences of Zimbabwean adolescents. This study adds to the knowledge base by investigating trauma symptom prevalence and association with resilience characteristics among a large school-based sample of rural and urban adolescents in Zimbabwe.

## Materials and methods

The aim of the overall study is to assess the impact of *Singing to the Lions* (www.crs.org/our-work/research-publications/singing-lions) a three-day school-based biopsychosocial workshop for trauma-affected youth in Zimbabwe, through a cluster randomized controlled trial. The present paper reports a cross-sectional analysis of the quantitative baseline data collected prior to intervention delivery, examining characteristics of trial participants as a large cohort of Zimbabwean children ages 11–18. We descriptively present and discuss children’s trauma symptomology by age, gender, and geography (rural versus urban) then examine relationships between trauma symptoms and resilience characteristics including self-esteem, self-efficacy, hope, self-calming, and social support.

### Study design and scope of present analysis

This program evaluation employed a two-phase, mixed-method, cluster randomized waitlist-controlled trial design. In Phase 1, schools (n = 13) were randomly assigned to either an immediate intervention condition or to a waitlist control condition by the community organization implementing the program. Quantitative data were collected by evaluators at three time points: baseline, post-intervention, and 3-month follow-up. Phase 2 employed participatory qualitative methods to assess effects of STL from the perspectives of students, caregivers, and teachers. Quantitative and qualitative data were collected between September 2024 and April 2025.

We hypothesized that trauma symptoms at baseline would be higher among rural youth in Chimanimani compared to urban youth due to the cumulative effects of both ongoing national stressors affecting Zimbabwean communities broadly and the additional exposure to disaster-related adversity following Cyclone Idai in 2019 [40,41]. It was also hypothesized that youth reporting more trauma symptoms would have lower levels of resilience characteristics.

### Study context

The study was conducted in two distinct communities, one rural and one urban—both characterized by high levels of poverty, high rates of trauma exposure, and extremely limited access to mental health services. Chimanimani is a rural community in Zimbabwe’s Eastern Highlands near the border of Mozambique where livelihoods are largely dependent on subsistence farming, forestry employment, and illegal gold mining. Health care in the area remains limited, despite efforts to improve access post-Cyclone Idai, and transport in the area is difficult often requiring people to walk long distances to school or medical facilities. In 2019, the area was severely damaged by Cyclone Idai and affected more than 270,000 people nationwide, many of whom required long-term humanitarian assistance and recovery support [42,43]. Today, the widespread community level trauma from this disaster is still evident, particularly when forecasted heavy rains raise collective fears of more landslides and loss of life. Children in Chimanimani were particularly impacted as many schools were converted to evacuation centers in the cyclone’s aftermath, and later, to shelters for displaced people [44]. Following the disaster, many schools in the district served as temporary evacuation centers and shelters for displaced families, disrupting children's education and normal routines. These prolonged school closures associated with the COVID-19 pandemic further interrupted learning for millions of Zimbabwean children and increased psychosocial vulnerabilities [45,46]. Soon after, schools were shut down due to the covid-19 pandemic, resulting in an estimated 1.5 missed school years. Since these crises, the government of Zimbabwe has acknowledged the great need for psychosocial support and community building programs in the area and has been supportive of development initiatives launched by non-governmental organizations in the region [44].

The high-density suburbs of Zimbabwe’s capital city of Harare are characterized by high population density, chronic infrastructural strain, and history of political violence [47,48]. Many of these neighborhoods have experienced massive population growth since the country’s independence but without proportional expansion of public services [30,47]. resulting in overcrowding, water and electricity insecurity, and inadequate sanitation—challenges that have been exacerbated by prolonged economic instability [49–51]. Studies using nationally representative data have documented high exposure to interpersonal violence for Zimbabwean children and adolescents and its association with poor health and wellbeing [15]. While research focused on Harare’s high-density contexts is limited, elevated community-level violence and intimidation that shapes young people’s safety and social environments have been documented [52].

### Local implementation partner

Founded in 2009, Miracle Missions Vimba (http://www.miraclemissionstrust.com/) is a non-political, non-profit organization run by Zimbabweans aiming to foster self-sufficiency in Zimbabweans offering a range of wellbeing and capacity building programs for children, older adults, women, people with disabilities, and prison populations. Miracle Missions had been delivering trauma-informed psychosocial support programs in Chimanimani since 2019 and throughout Harare’s high-density suburbs since Zimbabwe’s political violence of 2018. With support Miracle Missions implements *Singing to the Lions* in these two communities with a highly qualified team of Companions, resource persons, a psychologist, and educational consultants as well as team therapists.

### School and participant recruitment

Schools were recruited by the local implementation partner who met with education administrators to explain the intervention and purpose of the research then identify between 3–5 eligible classrooms at the school for participation based on students’ age, educational scheduling, and school capacity. Classroom level participation within each school was determined jointly by school administrators and the local implementing partner.

Consent procedures for participants were guided by protocols already in place for the organization which implemented Singing to the Lions workshops locally. Only children with both a signed parental consent form and a signed child assent form were eligible to participate in the evaluation (parents and/or children could also opt for the child to participate in the workshop but not the study assessments). The participant recruitment period for this study began on September 6, 2024 and ended on March 31, 2025.

### Parent trial allocation

Schools within each community were then stratified based on school characteristics (e.g., public or private) and demographic characteristics of the student body (e.g., poverty level) to identify one set of paired schools for each strata. The two paired schools were then randomly assigned to either the immediate intervention or waitlist control condition by the local implementation partner via video recorded coin toss. One intervention school opted out of the study after pre-test data were collected (but prior to the intervention being delivered) due to a scheduling conflict with other school activities, so an alternate school was randomly selected in its place. Randomization occurred as part of the parent trial and was not used in the present baseline analyses.

### Baseline data collection procedures

Trained enumerators conducted baseline assessments (T1) within one week prior to workshop delivery. To accommodate varying levels of literacy among youth respondents, questionnaires were administered in a private space outside the classroom. Prior to starting each questionnaire, the interviewer explained the Likert-type answer options using a visual printed on a sheet of paper so youth could point to their response along the 5-point scale. Interviewers would then ask two practice questions to ensure that young people understood the interview process and how to answer the questions posed.

When beginning data collection in a new classroom, the interviewer first obtained a class list showing the names of all students enrolled. This list was cross-referenced with signed consent/assent forms obtained previously, and any students without forms permitting collection of assessment data were crossed off the class list so they would not be interviewed. If a student with signed consent and assent forms in place was absent from school on a data collection day, the interviewer attempted to conduct the interview on a subsequent day. If a student with signed consent/assent forms was no longer attending that school (e.g., had transferred to a different school), the student was marked as “lost” on the interviewer’s class list and no attempt at further contact was made for the evaluation.

All data were collected via interviewer-administered questionnaire using Kobo Toolbox data collection app on tablet devices. Kobo Toolbox is an open source and customizable data collection platform designed for research in remote settings and widely used by humanitarian organizations and international development groups [53,54]. At the conclusion of each data collection day, the interviewer submitted all interview forms in the Kobo Toolbox app on their tablet, submitting data securely to cloud servers and wiping the data from memory on the tablet device. The local Research Coordinator monitored all data collection submissions on each interview day to confirm successful submission and ensure data quality to address any potential technological or user error issues quickly.

Youth participants in the intervention and control classrooms received a pack of school supplies (valued at $5) upon completion of the post-test assessment. Study protocols were approved by the Medical Research Council of Zimbabwe in addition to review by the Institutional Review Board at the Principal Investigator’s university.

### Measures

Study measures were identified, adapted, and translated through a participatory process between researchers and local collaborators which involved a series of video meetings and in-person work sessions over a two-month period. All measures were available in both Shona and English. Translation and backtranslation of instruments was conducted collaboratively with local translators with participation by the full data collection team in Zimbabwe. Psychosocial measures as well as demographic data (i.e., age, grade, gender, community) were assessed via interviewer-administered questionnaire.

#### Trauma symptoms.

To mitigate the risk of retraumatizing children by asking questions about traumatic events without treatment resources readily available [55], we assessed trauma symptoms rather than specific adverse childhood experiences. Children’s trauma symptomology was assessed using a locally tailored Trauma Symptoms scale created in Zambia.^35^ As part of a validation study, Murray and colleagues adapted the Post-traumatic Stress Disorder—Reaction Index (PTSD-RI) [30] by adding locally relevant items to the Trauma Symptoms Sub-scale then validated these alongside the original instrument using local responses to three cross-cultural criterion validity questions. The resulting 18-item culturally tailored symptoms sub-scale with locally relevant language demonstrated good discriminant validity and high internal consistency reliability (alpha = .94) [35]. This evaluation utilized the Zambian-modified and validated measure with a modified Likert-type response set to align with all other assessment measures as explained below (alpha = .87).

#### Resilience variables.

Instruments were identified and selected collaboratively by the Principal Investigator and local implementing partner who jointly prioritized selection of measures that were brief and either (a) standardized measures with acceptable reliability and validity which have been subsequently normed or used with youth in Sub-Saharan African contexts, or (b) designed specifically for use with the intervention.

*Self-Esteem*. The 10-item Rosenberg Self-Esteem Scale (RSES) is one of the most widely known measures of self-esteem, used in research from more than 50 countries [56]. The scale has been validated in numerous low- and middle-income countries and has demonstrated acceptable to high internal consistency reliability among African countries including Botswana, Ethiopia, Morocco, Tanzania, and Zimbabwe (alphas = 0.61 – 0.81) [57,58]. This study used the Child Version of the scale (CRSES) [59], which simplified question language for schoolchildren aged 7–12 years in 2021 and had been used in a study of South African youth [60] The CRSES’ Likert-type response scale was modified slightly for measure consistency across the evaluation (alpha = 0.72).

*Self-Efficacy*. The 24-item Self-Efficacy Questionnaire for Children (SEQ-C) [61] measures how young people cope with everyday problems and life stressors across three domains: social, academic, and emotional. Previous studies have reported high internal consistency reliability for both the overall scale and subscales (alphas ranging from 0.85 – 0.88) [61]. The SEQ-C has been used successfully in numerous studies with youth in South Africa [61,62], demonstrating high reliability and with content, construct, and face validity for South African youth established by a clinical psychologist [63]. This study used the Emotional Self-Efficacy subscale (alpha = 0.76) which included 8 items and a 5-point Likert-type response set, the anchors for which were modified to align with other study measures.

*Hope.* The Children's Hope Scale (CHS) [64] is the most frequently used measure to assess hopeful thinking in children and adolescents [65]. The CHS is intended for use with children ages 8–16 and includes 6 items to measure hope across two domains or subscales (agency and pathway) using a 6-point Likert-type response set. Research indicates that the CHS has high internal consistency reliability (subscale alphas = .83 -  .84) [66] and good test-retest reliability (subscale alphas = .71 and  .73) [64]. The CHS has demonstrated “good cross-cultural adaptation” in South Africa [66], good internal consistency reliability with South Sudanese youth displaced in Uganda (scale alpha = .79) [67], and good internal consistency reliability (subscale alphas = .75 and  .76) as well as strong test-retest reliability (*r* = .95) among conflict-affected children in Burundi [68]. The shortened 4-item version from the Healthy Youth Survey [69] which includes two items from each domain was used in this study with a modified 5-point Likert-type response set (alpha = 0.69).

*Self-Calming*. The *Singing to the Lions* intervention manual includes a series of suggested assessment questions intended to facilitate monitoring and evaluation efforts by implementing partners. While these suggested assessment items have not undergone psychometric testing, as with the Singing to the Lions program itself, the items were created by Zimbabwean mental health professionals and intended for use with Zimbabwean youth. The 4 items designed to assess children’s ability to regulate their emotions in stressful situations were used (e.g., *When I get scared, I am able to calm myself down;* alpha = 0.65 in the current study). The original trichotomous response set (Yes, No, Not sure) was modified to align with all other outcome measures.

*Social Support*. Four items from the *Singing to the Lions* intervention manual were used to assess children’s level of social support and support seeking behavior (e.g., *I know an adult in my community who I can talk to when I have a problem*). These items were created by Zimbabwean mental health professionals and had been used previously with Zimbabwean youth but had not undergone previous psychometric testing (alpha = 0.61 in the current study). The original 3-point response set was modified to align with other outcome measures.

#### Instrument adaptations.

The selected measures utilized response sets ranging from 4-point, 5-point, and 6-point Likert-type scales to trichotomous (yes, no, unsure) response sets. During the instrument selection process, community collaborators expressed concern that alternating between these highly divergent response formats across measures would confuse adolescent respondents, increase response error, and lengthen interview time placing additional burden on schools. Similarly, collaborators indicated that the agreement-based response anchors (e.g., “strongly agree”) used by some measures were less familiar and less easily interpreted than truth-based anchors (“not at all true” to “completely true”). Accordingly, the research team adopted a consistent five-point response format with locally understandable anchors. To further support participant comprehension of the response scale, children were provided a printed visual of five circles with varying portions filled in and a corresponding percent value below each [70] (e.g., the “Completely True” response option was illustrated by a completely darkened circle with text reading “100%” below it).

### Data analysis

Descriptive analyses were run to describe the sample and characteristics associated with trauma symptoms as well as most frequently endorsed trauma symptoms by gender. The prevalence of trauma symptoms was computed as the total scale score. Number of unique trauma symptoms endorsed was computed by dichotomously recoding scores for each scale item as 1 = Endorsed (i.e., youth answered “Somewhat True,” “Mostly True”, or “Completely True” for an item) or 0 = Not Endorsed (i.e., youth answered “Not at all true” or “A little true”). To compute the portion of youth who endorsed each symptom, the Likert-type scale responses to each of the 18 items were collapsed into binary indicators (1 = Endorsed; 0 = Not endorsed). Symptom experience was considered endorsed if the recorded response in the Likert-type scale was “Completely true,” “Mostly true,” or “Somewhat true.” Responses to the Likert-type scale of either “A little true” or “Not at all true” were considered not endorsing that symptom.

To examine differences in trauma symptomology by sociodemographic characteristics or implementation settings, we performed Pearson correlation, analysis of variance, or Pooled t-test, as appropriate. Pearson correlation was also used to examine associations between trauma symptoms and resilience characteristics. All analyses were performed under statistical significance of α = 0.05 using SAS v9.4 [71] and Stata SE 18 [72].

## Results

Participants (n = 1,342) ranged in age from 11-18 years ($\overset{―}{x}$=14.8, SD = 1.4). The sample included slightly more girls (54.6%) than boys and roughly equal proportions of children from rural (50.1%) and urban high-density (49.9) communities.; see Table 1.

**Table 1:** Participant characteristics (n = 1,342) at baseline.

	Range	$\overset{―}{x}$ (S.D.)
**Age**	11 - 18	14.83 (1.4)
	**n**	**%**
**Grade**		
Grade 6 (Primary)	32	2.4
Grade 7 (Primary)	49	3.7
Form 1 (Secondary)	268	20.0
Form 2 (Secondary)	414	30.9
Form 3 (Secondary)	321	23.9
Form 4 (Secondary)	57	4.25
Classrooms with mixed forms/grades	200	14.9
**Gender**		
Girls	733	54.62
Boys	609	45.38
**Community**		
Rural	675	50.15
Urban	669	49.85

Youth endorsed a wide range of trauma symptoms with an average of 7.6 symptoms (*SD* = 4.5) of 18 possible reported across the full sample. The most frequently reported symptom for both girls and boys was “I think too much” (endorsed by 61% of the full sample). Also highly endorsed by both girls and boys (53.4% of the full sample) was “I run if I see someone who hurt me.” Girls’ other most frequently reported symptoms were crying often, being easily startled, and being unable to open up. Among boys, the remaining most frequently endorsed symptoms were being always quiet, sleeping too much, and feeling shy (see Table 2).

**Table 2:** Trauma symptoms at baseline.

	Range	$\overset{―}{x}$ (S.D.)
Trauma Symptoms Scale Score	18-89	44.21 (13.7)
Total Trauma Symptoms Endorsed	0-18	7.6 (4.5)
	**n**	**%**
**Trauma Symptoms Endorsed**		
I think too much.	820	61.3
I run if I see someone who hurt me.	714	53.4
I am always quiet.	695	52.0
I cry often.	666	49.9
I am easily startled.	637	47.6
I feel shy.	636	47.5
I sleep too much.	624	46.6
I am reserved. I cannot open up.	596	44.5
I feel rejected, like everyone is against me.	549	41.1
I do not look like myself.	541	40.5
I do not feel at ease.	534	39.9
I am nervous.	526	39.3
I do not feel free.	526	39.3
I am damaged psychologically.	513	38.3
I have an unsettled mind, no peace of mind.	494	37.0
I am unhappy or sad.	483	36.1
I feel used.	342	25.6
I have stopped going to school because I think I will be laughed at or teased.	301	22.5
	**n**	**%**
**Trauma Symptoms by Gender**		
**Girls’ most frequently endorsed symptoms**		
I think too much.	456	34.1
I cry often.	424	31.7
I run if I see someone who hurt me.	417	31.2
I am easily startled.	377	28.2
I am reserved; I cannot open up.	355	26.5
**Boys’ most frequently endorsed symptoms**		
I think too much.	364	27.2
I am always quiet.	317	23.7
I run if I see someone who hurt me.	297	22.2
I sleep too much.	297	22.2
I feel shy.	282	21.1

Across the full sample, total trauma symptoms scale scores ranged from 18-89 (possible range: 18–90) with an average score of 44.21 (SD = 13.7). Total trauma symptom scores differed by gender with higher symptoms reported among girls (Mean = 45.4, SD = 14.0) compared to boys (Mean = 43.1, SD = 13.4; Pooled *t* = 2.9, *p* = 0.0039). However, there was no association between trauma symptomology and children’s age (*ρ* = -0.01, *p* = 0.7639), grade in school (*F*[6, 1262]=1.38, *p* = 0.2208), or community (i.e., rural or urban; Pooled *t* = 1.33, *p* = 0.1851); see Table 3.

**Table 3:** Associations between baseline characteristics and trauma symptomology.

Characteristic	*ρ*	p-value^1^
Age (years)	0.0023	0.9354
Hope	-0.08	**0.0034**
Self-calming	-0.31	**<0.0001**
Self-efficacy	-0.02	0.3490
Self-esteem	-0.36	**<0.0001**
Social support	-0.23	**<0.0001**

^1^Statistical significance of test evaluation association with trauma symptomology, Pearson correlation coefficient

Total trauma symptom scores were moderately negatively associated with resilience variables including self-calming (*ρ* = -0.31, p < 0.0001), self-esteem (*ρ* = -0.36, p < 0.0001), and social support (*ρ* = -0.23, p < 0.0001) with a weak negative association for hope (*ρ* = -0.08, p = 0.0034). However, trauma symptoms were not significantly associated with self-efficacy (*p* = 0.5118).

## Discussion

In this cross-sectional study, we examined the prevalence of trauma symptoms and their association with resilience characteristics among a school-based sample of adolescents in Zimbabwe. Overall, we found that reports of trauma symptoms were high with a mean score of 44.2 out of a possible range of 18–90. Youth reported more than 7 unique trauma symptoms on average, and some symptoms were endorsed by more than 60% of the full sample. We also found that trauma symptoms differed by gender—both in terms of prevalence (i.e., higher rates among girls compared to boys) and type (i.e., girls were more likely to report heightened sensitivity or emotional regulation responses while boys were more likely to report symptoms associated with being withdrawn). However, we found no significant differences in symptom prevalence based on youths’ age, grade, or community.

The high prevalence of trauma symptoms found in this study is consistent with much of the previous trauma research conducted in Sub-Saharan Africa. Chipalo found high rates of mental distress overall with moderate or severe psychological distress reported for more than a quarter of the Zimbabwean youth sampled [20]. A school-based study of South African adolescents found that nearly one-fourth of the young people reported symptoms consistent with a diagnosis of post-traumatic stress disorder [34], while a population-based study in Kenya reported PTSD rates of 18% among adolescents living in urban informal settlements [27].

However, our findings diverge from previous research in neighboring Zambia which produced the trauma symptoms measure used in our study. Murray and colleagues reported a mean trauma symptom scale score of 13.9 (SD = 16.5) out of a possible score range of 0–72 [35]. Using a scoring approach identical to that of the Murray study (i.e., adjusting symptom item scores to reflect a 0–4 response set rather than 1–5), the mean adjusted trauma symptoms score for Zimbabwean youth in our study was 26.2 (SD = 13.7)—nearly double that of the Zambian sample.

While the prevalence of trauma symptoms differed significantly between the current study and prior research from Zambia using the same trauma symptoms measure, the *types* of symptoms endorsed were highly consistent. In their study of 343 trauma-exposed children and adolescents from Zambia, Familiar and colleagues [36] found that the most frequently reported symptom was “I think too much”—which was also the most frequently reported symptom among Zimbabwean youth in the current study (endorsed by 54% and 61.3% of youth, respectively). Other highly endorsed trauma symptoms by both Zimbabwean and Zambian youth were being easily startled (53.1% and 47.6%, respectively) and sleeping too much (45.2% and 46.6%). The least endorsed trauma symptoms were also highly consistent across the two samples, including feeling used (17.8% and 25.6%) and avoiding school in fear of being laughed at or teased (16.6% and 22.5%). The only notable difference between these samples is that running away (i.e., “I run if I see someone who hurt me”) was reported by 61% of youth in our study while endorsed by only 29% of youth in the Zambia sample [36].

The prominence of “I think too much” among reported trauma symptoms is particularly salient in the Zimbabwean context. Kufungisisa (“thinking too much”) is a well-established Shona idiom of distress that encompasses emotional, psychological, and social suffering and is commonly used to describe experiences associated with depression, anxiety, and other common mental disorders [73]. The high endorsement of this symptom highlights the importance of culturally-specific trauma measures and suggests that Zimbabwean adolescents may express psychological distress more readily through locally meaningful concepts rather than through Western psychiatric constructs.

Previous research regarding associations between gender and trauma has been mixed. Consistent with our findings of higher trauma symptom rates among girls compared to boys, a study of South African adolescents by Suliman and colleagues found that girls reported significantly higher levels of PTSD symptoms as well as higher rates of depression and anxiety [34]. Conversely, studies of urban Kenyan youth and Zambian youth found no gender differences in PTSD or trauma symptoms, respectively [27,36]. Gender may also play a role in understanding the causes of trauma for Zimbabwean youth as boys and girls may be exposed to different forms of traumatic events. Although research in the Southern African region is extremely limited, studies from South Africa and Malawi suggest that boys are more likely to experience physical violence and assault while girls face higher risk of sexual violence [21,74]. Given that strong cultural and religious practices such as child marriage are a common feature in Zimbabwe which can lead to low self-esteem and self-efficacy for girls [75,76] more attention to the cultural and religious contexts of trauma is needed.

We hypothesized that trauma symptom prevalence would be higher for rural youth as a result of a natural disaster in 2019 that was associated with substantial community disruption and trauma exposure in Chimanimani and surrounding rural areas. However, study results indicate that trauma symptom levels were consistently high across both the urban and rural communities sampled. This contrasts with previous studies which found that context related differences influenced exposure to trauma [74,77] However, the high prevalence of adverse childhood experiences reported by a nationally representative sample of Zimbabwean young people [20] suggests that trauma exposure is relatively consistent regardless of geographic context. Moreover, the chronic political, climate, and economic conditions that have been prevalent in Zimbabwe and other Southern African countries, along with the exacerbating effect of covid-19, may contribute to ongoing psychosocial stressors among many youth [78–80].

Consistent with previous research finding that psychological resilience buffers trauma’s impact [81], our study found that trauma symptoms were inversely associated with resilience characteristics including social support, self-esteem, self-calming ability, and hope. Our results underscore the previously documented positive association between social support and mental health among young people in sub-Saharan Africa. In their study of former child soldiers in Uganda, Klasen and colleagues reported that lower perceived social support was linked with more severe trauma symptoms [82]. Similarly, a qualitative study with South African adolescents found that weak family support, loss of caregivers, and lack of adult guidance exacerbated psychological distress following trauma [83]. Studies of orphaned and vulnerable adolescents in Zimbabwe and South Africa further highlight social support as a contributor to mental health outcomes [84,85].

Several regional studies suggest that trauma exposure, particularly emotional and sexual violence, has a negative effect on adolescent self-esteem, but the relationship has often been examined indirectly. For example, nationally representative studies of adolescents and young adults in Zambia and Zimbabwe have found strong associations between violence in childhood and negative mental health outcomes such as psychological distress, depression, and suicidality while discussing diminished self-esteem or self-worth as a consequence of trauma exposure [38,39] Similarly, research on orphaned adolescents in Zimbabwe links trauma-related adversities to poor mental health outcomes while noting reduced self-esteem as a likely downstream effect [20].

While more limited, previous research in Sub-Saharan Africa linking trauma exposure to self-regulation in adolescents is consistent with our study findings on self-calming. In their study of former child soldiers in Uganda, Klasen and colleagues [82] found that chronic interpersonal trauma was associated with impairments in affective and behavioral regulation. Similarly, a study of South African youth identified self-calming as an important strategy for managing trauma-related stress [85].

Also limited is trauma research examining the construct of hope, despite its believed role in wellbeing, coping, and future orientation. One qualitative study conducted with South African adolescents found that cumulative trauma was associated with diminished hope and increased hopelessness [83]. Some studies included in a systematic review of trauma interventions for youth across sub-Saharan Africa reported improvements in hope alongside reductions in PTSD and depressive symptoms, although hope was rarely measured as an independent construct [86].

We had hypothesized an inverse relationship between trauma symptoms and each of the five resilience variables; however, trauma symptoms were not associated with self-efficacy. Previous research indicating that high exposure to adverse childhood experiences weakens an individual's coping capacity and increases the risk of long-term psychopathology would seem to support an expected inverse relationship between these constructs [22]. Moreover, concepts closely related to self-efficacy—including autonomy, choice, and control—are considered foundational principles of trauma-informed care in that they are believed to support trauma recovery [87].

Importantly, our study findings also align with observations made by professionals and lay professionals who work with children in Zimbabwe, such as the community partner for this study, Miracle Missions Vimba. Community practitioners have observed that many young people experience emotional, behavioral, and interpersonal difficulties that may reflect cumulative exposure to adversity but are not always recognized as trauma-related. The high prevalence of symptoms documented in this study provides empirical support for these practice-based observations and suggests that trauma-informed approaches are needed across sectors that regularly interact with children and adolescents, including education, medical care, social services, and disaster response.

The finding that trauma symptoms were highly prevalent in both the urban and rural communities sampled suggests that although the nature and sources of adversity may differ across geographic settings, youth in both types of settings may experience similar symptom burdens. Future research should examine how macro-level conditions interact with family, school, and community environments to influence trauma exposure, symptom expression, and access to protective resources. Exploring these multilevel processes could help distinguish which factors are relevant across Zimbabwe and which call for locally specific responses.

The high prevalence of trauma symptoms among students also has important implications for the Zimbabwean education system, where academic performance and discipline have traditionally received greater emphasis than learners’ mental health and psychosocial wellbeing. Trauma-related symptoms such as persistent rumination (“thinking too much”), hypervigilance, sleep disruption, emotional dysregulation, and social withdrawal may interfere with concentration, memory, classroom participation, and academic achievement. Trauma-informed educational practices may therefore help teachers and school administrators respond to student behavior with greater attention to its possible psychosocial context to improve student wellbeing and learning outcomes [88,89]. Teachers and support staff could be trained to recognize possible signs of distress, understand which practices could intensify trauma responses, and provide basic psychosocial support.

### Limitations and future recommendations

Several study limitations should be considered when interpreting our ﬁndings. First, while data were drawn from a larger randomized controlled trial, the results presented here utilizing baseline data are cross-sectional in nature. It is possible that higher trauma symptoms erode young people’s sense of hope, self-worth, capacity for self-regulation, and perceptions of available support or that the relationship is reciprocal, with trauma symptoms weakening resilience resources while reduced access to these resources makes it more difficult to cope with continuing adversity. Longitudinal research is needed to clarify the direction and development of these relationships over time. Second, we intentionally used a locally validated trauma symptoms measure from neighboring Zambia to provide a more culturally relevant approach to assessment rather than utilizing assessment tools generated and validated in Global North contexts [35]. While this approach brought greater regional cultural relevance, it is not known whether there may be differences in the ways that trauma symptoms are expressed by Zimbabwean young people compared to Zambian youth. Third, to improve participant comprehension, several resilience measures were administered using harmonized response formats and culturally adapted response anchors developed collaboratively with local partners. Our efforts were guided by research emphasizing that culturally appropriate measurement adaptation extends beyond item translation to include response formats and other features of instrument administration when necessary to improve conceptual equivalence and local validity [70,90,91]. Nevertheless, these adaptations reduce direct comparability with studies using the original response formats and should be considered when interpreting findings. Finally, the geographic boundaries of our sample should be considered. While the inclusion of one rural community (Chimanimani) and one urban and peri-urban community (Harare) allowed us to sample a wide range of public and private schools representing a wide range of economic and educational contexts, our sample was not representative and thus results may not be generalizable to all school-aged youth across Zimbabwe.

Despite these limitations, this study helps to extend a limited body of research on trauma symptoms and resilience among youth in Zimbabwe and adds to growing evidence of the need for culturally and contextually appropriate responses. Existing intervention studies from Sub-Saharan Africa offer several lessons for the development of these responses. Promising programs have typically adapted intervention language and delivery formats, incorporated locally relevant concepts, used group-based methods, and trained lay providers to deliver services with ongoing supervision. Examples include Pamoja Tunaweza (“Together We Can”), a group-based trauma-focused cognitive behavioral therapy program implemented in Kenya and Tanzania using Kiswahili and lay counselors; a culturally adapted TF-CBT intervention in Zambia delivered to orphaned and vulnerable children by trained lay counselors; and the Youth Readiness Intervention in Sierra Leone, which combined psychoeducation and cognitive restructuring with self-regulation, communication, and problem-solving skills for war-affected youth. The Kenya and Tanzania program produced greater short-term improvement than usual care at three of four sites, although effects were less consistent at 12-month follow-up [92], while the Zambian intervention significantly reduced trauma symptoms relative to the control condition [93]. The Sierra Leone intervention did not significantly reduce psychological distress or post-traumatic stress symptoms but improved emotional regulation, prosocial behavior, and functional impairment [94].

Taken together, these studies suggest that culturally adapted, task-shared interventions may expand access in settings with limited mental health services and that intervention outcomes should include functioning and coping as well as symptom reduction. They also highlight the need for approaches to cultural adaptation of interventions to extend beyond translating intervention materials to incorporate local conceptualizations of distress, address how local mental health services are typically provided, and how families and communities wish to be involved.

Several directions for future research emerge from the current study findings. First, studies are needed to develop and validate culturally grounded assessment tools for Zimbabwean and Southern African youth. While the use of a trauma symptoms measure adapted in neighboring Zambia represents an important step [35], further research is needed to examine whether the measure captures the full range of Zimbabwean adolescents’ experiences. This work should include locally meaningful idioms of distress [95] such as *kufungisisa* (“thinking too much”) [73], while also examining whether symptom meanings differ by individual or community characteristics or type of trauma exposure. Participatory instrument development approaches could lead to development of new measures that are rooted in how young people and communities understand psychological distress rather than adapting measures based on Western diagnostic categories.

Second, longitudinal research is needed to understand how trauma symptoms and resilience change over time in the Zimbabwean context. Studies should explore whether resilience characteristics such as social support, self-esteem, self-calming, and hope predict reductions in symptoms, whether increased trauma symptoms hamper these resilience resources, or whether the relationships are bidirectional. Longitudinal studies could also identify developmental periods when Zimbabwean youth are particularly vulnerable and determine whether protective factors function differently across developmental stages.

Third, future studies should explore the types, timing, and cumulative effects of trauma exposure more directly. While this study examined trauma symptoms, it did not assess which specific adverse childhood experiences caused those symptoms. Research exploring exposure to chronic stressors within the Zimbabwean context such as economic instability, food insecurity, community displacement, natural disasters, and political violence could help to identify which forms of adversity contribute to particular symptom patterns or resilience profiles. This research could also help explain why trauma symptom prevalence was similarly high across the rural and urban communities in the current study despite differences in their histories and social environments.

Finally, research is needed to inform implementation of Zimbabwe’s Mental Health Strategy in order to integrate mental health services with educational, medical, and social services. Help-seeking among Zimbabwean youth remains low [96], with barriers including stigma, limited knowledge of services, self-blame, fear of victimization, and cultural expectations surrounding emotional expression [97–99]. Studies indicate that 75% of Zimbabweans seek both traditional and biomedical mental health care [100], suggesting that service models based on Western clinical systems do not reflect existing patterns of help-seeking. Thus, research should examine how schools, primary care providers, community organizations, traditional healers, and formal mental health professionals could uniquely contribute to coordinated and culturally responsive systems of support.

## Conclusion

This study contributes to the limited knowledge base around child and adolescent mental health in Zimbabwe. The findings support continued development and evaluation of culturally grounded, trauma-informed approaches that are feasible in Zimbabwean communities with limited or no mental health services. Such approaches could include strengthening the ability of teachers and other adults to recognize trauma-related distress and developing interventions that draw upon local language, community-based knowledge, and existing systems of care. Longitudinal and intervention research are needed to determine how trauma symptoms develop over time, which resilience characteristics are protective across children’s developmental stages, and which models of psychosocial support are effective, sustainable, and acceptable to trauma-affected Zimbabwean young people and their communities.

## Supporting information

S1 Checklist PLOS inclusivity in global research checklist. (PDF)
